# Prevalence of hemorrhagic fever with renal syndrome in Qingdao City, China, 2010–2014

**DOI:** 10.1038/srep36081

**Published:** 2016-10-27

**Authors:** Fachun Jiang, Zhentang Zhang, Liyan Dong, Bi Hao, Zaifeng Xue, Dongqiang Ma, Hang Su, Hong-ling Wen, Hao Yu, Xue-jie Yu

**Affiliations:** 1 Qingdao City Center for Disease Control and Prevention, Qingdao City, Shandong Province, 266033, China; 2 Huangdao District Center for Disease Control and Prevention, Qingdao City, Shandong Province, 266400, China; 3 School of Public Health, Shandong University, Jinan, Shandong Province, 250012, China; 4 School of Medicine, Fudan University, Shanghai, 200433, China; 5 Department of Pathology, University of Texas Medical Branch, 301 University Blvd, Galveston, TX 77555-0609, USA.

## Abstract

Hemorrhagic fever with renal syndrome (HFRS) was considered to be transmitted by *Apodemus agrarius* and *Rattus norvegicus*, the principal animal hosts of Hantaan virus and Seoul virus, respectively. The aim of this study is to determine the correlation of HFRS incidence with capture rate and hantavirus infection rate of rodent species in Qingdao City, China. We collected HFRS patients’ information and captured field and residential rodents in Qingdao City, China from 2010 to 2014. The correlations of HFRS incidence to rodent capture rate and hantavirus infection rate of rodents were analyzed statistically. The main findings of this study are that the high HFRS incidence (19.3/100,000) is correlated to the capture rate of field *Mus musculus* (p = 0.011, r = 0.037); but surprisingly it did not correlated to the capture rate of the principal rodent hosts *Apodemus agrarius* and *Rattus norvegicus* and the hantavirus infection rate of these rodent species in the field or residential area. These novel findings suggest that *Mus musculus,* a nontraditional animal host of hantavirus may play an important role in hantavirus transmission in Qingdao City.

Hantavirus infections occur worldwide, but approximately 90% of hemorrhagic fever with renal syndrome (HFRS) cases has been reported in China^1^. During 2006–2012, a total of 77,558 HFRS cases have been reported in China with 866 deaths^2^. Qingdao City located in eastern China is one of multiple high incidence areas of HFRS in China^3^. Due to its wide geographic distribution, Hantavirus has evolved into different serotypes in different areas of the world^4^. In China, HFRS is caused by *Hantaan* virus (HTNV) and Seoul virus (SEOV)^4^,^5^,^6^. The principal rodent host of HTNV is the striped field mouse *Apodemus agrarius*, and the principal rodent host of SEOV is commensal rodent brown rat *Rattus norvegicus*^7^,^8^. The aim of this study is to determine the correlations of HFRS incidence to rodent species and to hantavirus infection rate in the rodent species in Qingdao City from 2010 to 2014.

## Results

### HFRS cases in Huangdao District of Qingdao City

**Figure 1:**
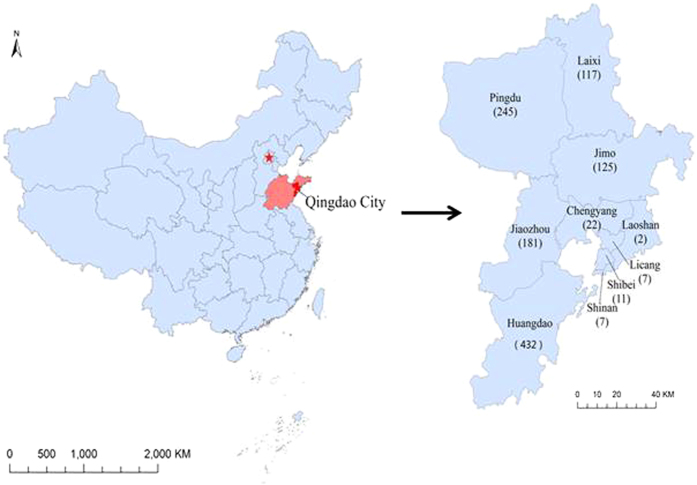
Geographic location of Huangdao District in Qingdao City, China. On the left is the map of China, in Which Qingdao City was marked as dark. On the right is the map of Qingdao City with Huangdao district. The maps were constructed using ArcGIS10.1 software (http://resources.arcgis.com/en/home/).

**Figure 2:**
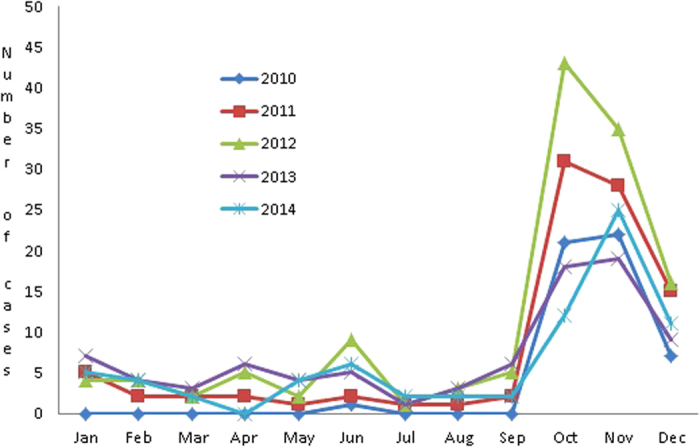
Monthly distribution of HFRS cases in Huangdao District of Qingdao City from 2010 to 2014.

A total of 432 HFRS patients were diagnosed in Huangdao District with 51, 92, 129, 85 and 75 cases, respectively in each year from 2010 to 2014 (Fig. 1). The incidence of HFRS in rural population was 11.4, 20.5, 28.9, 19 and 16.7 every 100,000 population, respectively in each year from 2010 to 2014. The highest prevalence of HFRS occurred in 2012. The profile of HFRS prevalence was similar each year. A low level of HFRS prevalence occurred from January to September each year; a small peak of HFRS prevalence occurred in the spring and summer between April and June in some years; and a high peak of HFRS prevalence occurred each year between October and December (Fig. 2).

All 432 patients were from rural areas and 86.7% (379/432) of patients were farmers. Of the patients, 97.5% (421/432) had exposure history to rodents within 3 months before onset of illness. The sex ratio of the patients was 2.5 to 1 (308 male to 124 female). The age of the patients ranged from 5 to 85 years old with median age of 50 years old. Majority of the patients (85.4%, 369/432) occurred between 31–70 years old and more than half (56.9%, 246/432) occurred between 41 and 60 years old.

### Correlation between HFRS incidence and annual rodent capture rate

The captured animals included rodents and shrews. Only rodents were analyzed for correlation with the incidence of HFRS. The rodent capture rate was 1.6% to 7.1% in field areas and 1.4% to 5.1% in residential areas (Table 1). The captured rodent species in the order of average annual capture rate from high to low were *Apodemus agrarius* (1.6%), *Rattus norvegicus* (1.0%), *Mus musculus* (0.8%), and *Rattus rattus* (0.3%) in the field area and were *Rattus norvegicus* (1.4%), *Mus musculus* (1.4%), and *Rattus rattus* (0.2%) in the residential area (Table 1). The rodent species and density varied in different years with the peak in 2012 (Table 1) for both field and residential rodents. The capture rate of rodents increased from 2010 to 2012 and declined from 2012 to 2014 annually (Table 1). *Mus musculus* and *Rattus norvegicus* were consistently present at a high frequency in both field and residential areas; *Rattus rattus* had relatively high frequency in both residential and field areas in 2010 and 2011, but disappeared from both areas from 2012 to 2014. *Apodemus agrarius* was not captured in 2010 and 2011, but started to appear in high numbers in the fall of 2012 and decreased in the summer of 2014 in the field (Table 1). *Apodemus agrarius* was not captured in residential areas in 2010 and 2011 and very few *Apodemus agrarius* were captured from 2012 to 2014.

**Figure 3:**
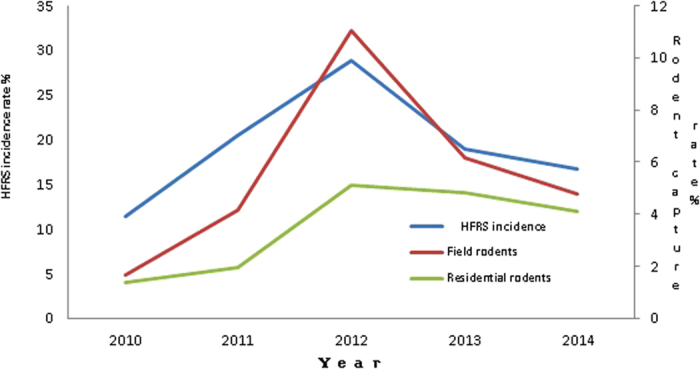
Comparison of human HFRS incidence and rodent capture rate in Huangdao District, 2010–2014. The scale on the left indicated HFRS incidence of 100,000 populations and the scale on the right indicated the annual capture rate of field rodents and residential rodents.

Five-year surveillance indicated that the annual incidence of HFRS and annual rodent capture rate had the same profile (Table 1 and Fig. 3). When rodent capture rate was high, HFRS incidence was also high, and vice versa. Both rodent capture rate and HFRS had a peak in 2012. Statistical analysis with Pearson Correlation showed that the incidence of HFRS from 2010 to 2014 was correlated to the annual capture rate of field rodents (p = 0.018, r = 0.938), but not the capture rate of residential rodents. For individual species of the rodents, the HFRS incidence was correlated to the annual capture rate of *Mus musculus* from the field (p = 0.011, r = 0.037), but was neither correlated to the annual capture rate of other field rodent species nor all residential rodent species from 2010 to 2014.

### Correlation between HFRS incidence and quarterly rodent capture rate

**Figure 4:**
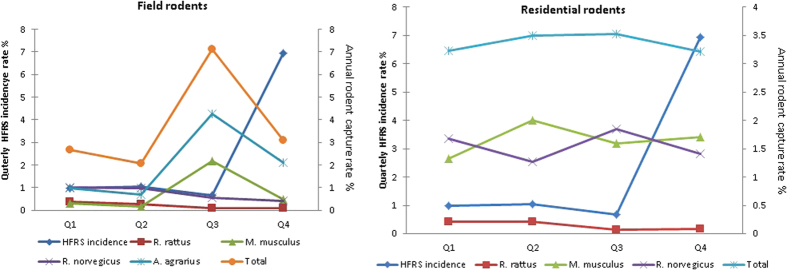
Comparison of quarterly HFRS incidence and quarterly rodent capture rate in Huangdao District, 2010–2014. The scale on the left indicated quarterly HFRS incidence of 100,000 population and the scale on the right indicated the quarterly capture rate of field rodents and residential rodents.

Because rodents were captured quarterly, we further analyzed the correction between the quarterly incidence of HFRS and the quarterly capture rate of rodents. The quarterly capture rate of field rodents varied from 2.7% to 7.1% in different quarters. The peak capture rate occurred in the third quarter and the next highest capture rate was in the fourth quarter. Both *Apodemus agrarius* and *Mus musculus* had a peak capture rate in the third quarter (Fig. 4). The quarterly capture rate of residential rodents changed little year-round, and ranged from 3.2% to 3.5% with average capture rate of 3.4% (Fig. 4).

Statistical analysis indicated that the incidence of HFRS was not correlated to quarterly capture rate of field rodents, residential rodents, or any individual species of rodents from field or residential areas. However, the incidence of HFRS in each quarter was correlated to the capture rate of *Apodemus agrarius* (p = 0.014, R = 1) and *Mus musculus* (p = 0.000, r = 1) in the previous quarter.

### Hantavirus infection rate in rodents

Detection of hantavirus antigen in the lungs of rodents with hantavirus specific monoclonal antibody by immunofluorescence assay showed that the 5-year infection rate of hantavirus was 1.9% (1/54) to 3.8% (9/240) for residential rodents and 0.9% (1/113) to 6.9% (25/364) for field rodents between 2010 and 2014 (Table 2). The 5-year average infection rate of field rodents (5.7%, 59/1041) was higher than those of the residential rodents (3.4%, 35/1035). Except for one year, the infection rate of field rodents was always higher than residential rodents (Table 2). Statistical analysis showed that the HFRS incidence did not correlate to the annual infection rate of any rodent species.

Analysis of the hantavirus infection rate of rodents in each quarter indicated that all rodent species from field and residential areas were infected year-round with the highest infection rate in the first quarter and the second highest infection rate in the fourth quarter for both field and residential rodents (Table 3). Statistical analysis showed that the HFRS incidence rate did not correlated to the quarterly infection rate of any rodent species.

## Discussion

The incidence of HFRS in Huangdao District is very high, ranged from 11.4 /100,000 to 28.9/100,000 from 2010 to 2014. The epidemiology of HFRS from 2010 to 2014 indicated that there are two peaks of HFRS cases in Huangdao District of Qingdao City. A small peak occurred in the spring to summer seasons and a large peak occurred in the fall season between September and December. Previous studies indicated that the spring peek is caused by Seoul virus and the autumn peak is caused by *Hantaan* virus^3^.

Human pathogenic hantaviruses are transmitted by rodents^8^. After infection with hantavirus, rodents carried hantavirus and excreted hantavirus for a long period. In hantavirus infected *Apodemus agrarius*, the virus has been detected in the blood from 7 to 12 days, in saliva 9–40 days, in urine 9–360 days, in feces 12–40 days, in lung 12–180 days, in kidney 15–18 days, and in liver 12–40 days^9^,^10^. Therefore, rodent species distribution, density, and hantavirus infection rate are the main factors affecting the incidence rate of HFRS in an area. HFRS is caused by *Hantaan* virus and Seoul virus in China and currently these viruses were recognized to be transmitted by *Apodemus agrarius* and *Rattus norvegicus*, respectively.

We captured rodents from 2010 to 2014 in Huangdao District, a high HFRS incidence area to determine the correlation between rodent species and HFRS incidence. The five-year surveillance indicated that density of field rodent population has dramatically changed in different seasons with a low density in the first two quarter (January to June), a rapid rise in the third quarter (July to September), and a fall in the fourth quarter (October to December). The seasonal changes of field rodent population density may be caused by the availability of food and weather condition. The density of residential rodents changed little year-round, suggesting that the residential rodents have a stable food source and weather condition at all the time. *Apodemus agrarius* and *Mus musculus* are the dominant species of the field rodents. Both of them have a peak population in the third quarter, resulting in a third quarter rise of field rodent population. The major residential rodents are *Mus musculus* and *Rattus norvegicus*. Striped field mouse *Apodemus agrarius* is rarely captured in residential areas, however, commensal rodents *Rattus norvegicus*, *Rattus rattus*, and *Mus musculus* have similar density in field areas and residential areas, suggesting that *Apodemus agrarius* mainly lives in field areas and *Rattus norvegicus*, *Rattus rattus*, and *Mus musculus* live in both residential areas and field areas.

According to the rodent transmission model of hantavirus, the density and hantavirus infection rate of principal rodent hosts will determine the HFRS incidence in an area with a stable human population. Our study indicated the annual capture rate of field rodents and residential rodents in Huangdao District had the same tendency with the annual incidence rate of HFRS. HFRS annual incidence rate is correlated to the density of field rodents, but not the density of residential rodents, suggesting that the high HFRS incidence is caused by high density of field rodent population. *Apodemus agrarius* is considered as the principal rodent host of *Hantaan* virus in China. However, in 2010 and 2011, the HFRS incidence was very higher in the fall season even though *Apodemus agrarius* was not captured during this time. It suggests that *Hantaan* was transmitted by other rodent species in 2010 and 2011 when *Apodemus agrarius* was absent. Statistical analysis indicated that HFRS incidence correlated to the density of *Mus musculus* in the field area, but not *Apodemus agrarius* from field. It is surprising that the annual capture rate of *Apodemus agrarius*, the principal animal host of *Hantaan* virus in China, is not correlated to the incidence of HFRS. The density of rodent species in the field and residential areas has changed dramatically in different years. *Apodemus agrarius* was not captured in 2010 and 2011, but had a high density after 2012; in contrast, *Rattus rattus* had a relatively high density in the 2010 and 2011 and disappeared since 2012. We do not know what cause the fluctuation of these rodent species in the field and residential areas.

*Mus musculus* was not considered a rodent host of *Hantavirus* until recently^11^. Previous studies have demonstrated that *Mus musculus* carries both Seoul virus and *Hantaan* virus. We have detected *Hantaan* virus RNA in *Mus musculus* captured in field in Huangdao District previously^11^. Seoul virus RNA was detected in *Mus musculus* captured on residence area in Shandong, Hunan, Beijing and Inner Mongolia in China^12^,^13^,^14^,^15^; *Mus musculus* was antibody positive to hantavirus (9.1%) in South Korea^16^. *Mus musculus* might also play an important role in harboring and transmitting PUU-like viruses in Europe^17^,^18^. Our study indicated that *Mus musculus* has a high density in field and residential areas and a high infection rate with hantavirus. We also demonstrated that both the annual and quarterly capture rates of *Mus musculus* are correlated to the incidence of HFRS. Our study suggests that *Mus musculus* is important in the transmission of hantavirus to humans in China.

The quarterly HFRS incidence is statistically correlated with the capture rate of *Apodemus agrarius* and *Mus musculus* in the previous quarter. This delayed rodent density-dependent incidence of HFRS needs to be further investigated by capturing rodents monthly in a future study.

The highest hantavirus infection rate of rodents was in March, but the highest HFRS incidence was in October. Statistical analysis indicated that HFRS incidence is not correlated to the infection rate of either field rodents or residential rodents. The fluctuation of rodent infection rate of hantavirus may be not sufficient to affect the incidence of HFRS in humans. Our result suggests that the density of rodent populations rather than hantavirus infection rate of rodents in Huangdao District is more important in transmission of hantavirus. With high density of field rodents, people may have increased chance to contact infected rodents, resulting in a higher risk of infection with hantavirus.

In conclusion, our study indicates that *Mus musculus* play an important role in hantavirus transmission in Qingdao City, China.

## Materials and Methods

### Ethics statement

The ethical committee of the Qingdao Center for Disease Control and Prevention has approved all human and animal works and the study was carried out according to the medical research regulations of China. The informed consent was obtained from all patients.

### Study site

We collected rodents in rural areas of Huangdao District of Qingdao City in Shandong Province of China located at longitude 119°30′–120°30′ and latitude 35°35′–36°08′. The district consists of low-lying hills with forests and farmlands, which is the typical niche for the presence of *Hantavirus* animal hosts. The rodents were captured in the same villages, but in different locations of each village every year to avoid disturbing the local rodent density, species, and hantavirus infection rate. The population of the Huangdao district was 843,276 with rural population 447,837 in 2012 (http://baike.baidu.com/view/49758.htm?fromtitle=%E8%83%B6%E5%8D%97%E5%8E%BF&fromid=8061645&type=syn).

### HFRS patients

The data of HFRS patients in Qingdao City from January, 1 to December 31 from 2010 to 2014 was collected from the national infectious diseases surveillance and reporting system (http://1.202.129.170). Suspected HFRS patients were diagnosed according to the criteria in the < Diagnostic criteria for epidemic hemorrhagic fever WS278-2008> issued by the Ministry of Health of China in 2008^19^.

### *Hantavirus* reservoir animal surveillance

Rodents were trapped once each quarter in March, June, September, and November, respectively with the first quarter starting in January. Rodents were trapped using snap-traps with peanut bait from 2010 to 2014. The traps were set before sunset inside farmhouses or farmyards (residential areas) and in farmlands 500 meters away from farmhouses (field areas). Two or three snap-traps were set in each residential area and the distance between traps was 10 meters in the field. The trapped animals were collected in the morning and their species identified morphologically. Rodent capture rate was determined by dividing the number of trapped rodents by the number of traps used each year.

### Detection of *Hantavirus* antigens

Rodent lung tissues were harvested aseptically and frozen at −70 °C. Viral antigens in the rodent lung tissues were detected by using indirect immunofluorescence assay (IFA) with anti-*Hantavirus* monoclonal antibody reacting with both HTNV and SEOV antigens as described previously^20^.

### Statistical analysis

Statistical analysis of correlation between HFRS incidence, rodent capture rate and infection rate was performed with Pearson Correlation, SPSS16.0.

## Additional Information

**Publisher's note**: Springer Nature remains neutral with regard to jurisdictional claims in published maps and institutional affiliations.

**How to cite this article**: Jiang, F. *et al.* Prevalence of hemorrhagic fever with renal syndrome in Qingdao city, China, 2010–2014. *Sci. Rep.* **6**, 36081; doi: 10.1038/srep36081 (2016).

## Figures and Tables

**Table 1:** Comparison of annual human HFRS incidence and rodent capture rate in Huangdao District, 2010–2014.

Year	HFRS incidence	Capture rate of field rodents (%)					Capture rate of residential rodents (%)				
		*Apodemus agrarius*	*Rattus norvegicus*	*Rattus rattus*	Mus musculus*	Total*	*Apodemus agrarius*	*Rattus norvegicus*	*Rattus rattus*	*Mus musculus*	Total
2010	11.4	0	0.9	0.5	0.2	1.6	0	0.7	0.2	0.5	1.4
2011	20.5	0	2.1	1.2	0.9	4.2	0	0.9	0.65	0.4	2
2012	28.8	3.7	1.5	0	1.9	7.1	0.04	2.3	0	2.1	5.1
2013	19	2.5	0.2	0	0.5	3.2	0.04	1.7	0	2.1	4.8
2014	16.7	1.7	0.3	0	0.7	2.7	0.02	1.5	0	2.1	4.1
Average	19.3	1.6	1	0.3	0.8	3.8	0.02	1.4	0.2	1.4	3.5

Rodent snaps in each year were 5427, 2758, 5228, 11383, 4191 for 2010, 2011, 2012, 2013 and 2014, respectively. The capture rate each year was calculated by the number of rodents captured/snap number × 100.

^*^Indicated that a significant correlation between HFRS incidence rate and the total capture rate of field rodents (r = 0.938, P = 0.018) and the capture rate of *Mus musculus* (r = 0.037, p = 0.011).

**Table 2:** Annual hantavirus infection rate in rodents in Huangdao District from 2010 to 2014.

Year	Field rodent infection rate (%)					Residential rodent infection rate (%)				
	*A. agrarius*	*M. musculus*	*R. norvegicus*	*R. rattus*	Total	*A. agrarius*	*M. musculus*	*R. norvegicus*	*R.rattus*	Total
2010	0 (0/0)	0 (0/12)	4 (2/50)	11.1 (3/27)	5.6 (5/89)	0 (0/0)	0 (0/26)	2.9 (1/34)	3.7 (1 /27)	2.3 (2/87)
2011	0 (0/0)	0 (0/22)	0 (0/59)	3.1 (1/32)	0.9 (1/113)	0 (0/0)	0 (0/11)	4 (1/25)	0 (0/18)	1.9 (1/54)
2012	6.8 (13/191)	7.2 (7/97)	6.6 (5/76)	0 (0/0)	6.9 (25/364)	0 (0/2)	2.6 (3/115)	4.9 (6/123)	0 (0/0)	3.8 (9/240)
2013	3.4 (10/290)	7.5 (4/53))	35 (7/20)	0 (0/0)	5.8 (21/363)	0 (0/4)	2.2 (6/271)	5.4 (12/224)	0 (0/0)	3.6 (18/499)
2014	5.5 (4/73)	11.1 (3/27)	0 (0/12)	0 (0/0)	6.3 (7/112)	0 (0/1)	1.1 (1/91)	6.3 (4/63)	0 (0/0)	3.2 (5/155)
Total	4.9 (27/554)	6.6 (14/211)	6.5 (14/217)	6.8 (4/59)	5.7 (59/1041)	0 (0/7)	1.9 (10/514)	5.1 (24/469)	2.2 (1/45)	3.4 (35/1035)

**Table 3:** Quarterly hantavirus infection rate in rodents in Huangdao District from 2010 to 2014.

Quarter	Field rodent infection rate (%)					Residential rodent infection rate %				
	*A. agrarius*	*M. musculus*	*R. norvegicus*	*R. rattus*	Total	*A. agrarius*	*M. musculus*	*R. norvegicus*	*R. rattus*	Total
Q1	7.2 (5/69)	19.0 (4/21)	5.6 (4/72)	15.4 (4/26)	9.0 (17/188)	0 (0/0)	3.1 (3/98)	8.1 (10/123)	6.3 (1/16)	6.3 (14/237)
Q2	1.9 (1/54)	7.1 (1/14)	3.9 (3/76)	0 (0/20)	3.0 (5/164)	0 (0/0)	0.6 (1/174)	1.8 (2/111	0 (0/19)	1.0 (3/304)
Q3	4.4 (12/270)	4.3 (6/139)	5.6 (2/36)	0 (0/6)	4.4 (20/451)	0 (0/1)	1.9 (2/108)	3.2 (4/125)	0 (0/3)	2.5 (6/237)
Q4	5.6 (9/161)	8.1 (3/37)	15.2 (5/33)	0 (0/7)	7.1 (17/238)	0 (0/6)	3.0 (4/134)	7.3 (8/110)	0 (0/7)	4.7 (12/257
Total	4.9 (27/554)	6.6 (14/211)	6.5 (14/217)	6.8 (4/59)	5.7 (59/1041)	0 (0/7)	1.9 (10/514)	5.1 (24/469)	2.2 (1/45)	3.4 (35/1035)

Q1, 2, 3 and 4 indicate Quarter 1, 2, 3 and 4, respectively.
